# Genomic signatures of selection reveal genetic mechanisms underlying economic traits in Licha black pigs

**DOI:** 10.5713/ab.250712

**Published:** 2025-12-03

**Authors:** Jiajia Liu, Zhe Tian, Mubin Yu, Wenwen Li, Pengcheng Lv, Tao Wang, Yu Tian, Shuer Zhang, Junjie Wang, Wei Shen

**Affiliations:** 1 Key Laboratory of Animal Reproduction and Biotechnology in Universities of Shandong, College of Animal Science and Technology, Qingdao Agricultural University, Qingdao, China; 2 General Station of Animal Husbandry of Shandong Province, Jinan, China; 3 Protection of Animal Genetic Resources and Biological Breeding Engineering Research Centre of Shandong Province, Jinan, China

**Keywords:** Economic Traits, Licha Black Pig, Selection Signature, *Sus scrofa*, Whole-genome Sequencing

## Abstract

**Objective:**

The Licha black pig (LC) is a Chinese indigenous breed that is nationally protected, recognized for its superior meat quality and strong environmental adaptability. However, the population has experienced a rapid decline due to extensive crossbreeding with commercial lines. Understanding the genetic basis of economically important traits is crucial for conservation and genomic improvement.

**Methods:**

Whole-genome resequencing was performed on 120 LC pigs and combined with genomic data from 285 pigs representing 32 global populations, including wild boars, commercial breeds, and other Chinese indigenous pigs. Population structure was investigated using phylogenetic trees, principal component analysis, ADMIXTURE and TreeMix analysis. Selection signatures were identified through four complementary approaches (FST, θπ ratio, XP-CLR, and Tajima’s D). Candidate genes were examined through functional enrichment analysis, protein structure prediction, and cross-referencing with trait association and tissue-specific expression databases. Phenotypic data on body size and teat number were also collected in LC pigs for targeted genotype–phenotype analysis.

**Results:**

Phylogenetic analyses showed clear stratification among global pig populations, with Chinese indigenous breeds significantly separated by the Qinling-Huaihe Line. LC pigs formed a distinct genetic cluster between northern Chinese and European breeds. Selective sweep analyses identified several candidate genes under positive selection, including *SOCS6* and *ATP2B4* (skeletal muscle development), *RASAL2* (adipogenesis), and *DOCK2* (male fertility). Trait-focused analyses identified *ZNRF3* as a major locus for body size, with a missense mutation (g.46228935G>A; Gln→Arg) predicted to influence Wnt/β-catenin signaling. Signals of selection in *ADGRB3*, a gene potentially involved in teat patterning and mammary gland vascularization, were associated with variation in teat number.

**Conclusion:**

Our genomic analyses provide new insights into the genetic architecture of economically important traits and environmental adaptation in the LC. These findings provide a foundation for genomic-informed selective breeding and present valuable molecular tools for the genetic improvement and sustainable utilization of this indigenous genetic resource.

## INTRODUCTION

The Licha black pig (LC), an indigenous breed from Shandong Province, China, is highly valued for its adaptability, fertility, and tolerance to coarse feed, earning it the reputation “Taihu in the South, Licha in the North” [1]. A distinctive characteristic of this breed is the presence of one or two additional thoracic–lumbar vertebrae, which extend carcass length and enhance meat yield - traits of considerable economic importance [2]. Owing to these advantages, LC has been recognized as a National Key Protected Livestock Genetic Resource since 2011. Nevertheless, its population has declined markedly since the 1940s due to extensive introgression from commercial breeds such as Landrace and Yorkshire. Purebred numbers have decreased by more than 40% since 2000, and the effective population size (Ne) is currently estimated at only 8.7, coupled with moderate genomic inbreeding. These trends signal ongoing genetic erosion and highlight the urgent need for conservation [3].

Previous studies have identified candidate genes associated with key economic traits in LC pigs. Notably, *NR6A1* has been established as a major regulator of vertebral number and body length in pigs and other vertebrates [4]. Xie et al [2] further confirmed its role in LC using single nucleotide polymorphisms (SNPs) chip and resequencing data, identifying a likely introgressed allele at chromosome 1:26534726 (A) derived from commercial breeds. Additional genes, including *LRP5, SP5*, and *LRP8* have also been implicated in vertebral development [5]. In reproductive performance, beneficial alleles in *CEBPB* and *FSHR*, which influence follicle development and hormone signaling, have been linked to increased fertility [6]. Regarding meat quality traits, interactions among *NR6A1, PAPPA2*, and *PIK3C2B* appear to regulate *FASN*, a gene central to intramuscular fat deposition and fatty acid composition [7]. Moreover, *CAST* has been proposed as an important determinant of meat tenderness and related traits [8]. While these findings provide valuable insights, they are largely trait-specific and do not comprehensively address the evolutionary processes or selective pressures shaping LC genomes. Systematic genome-wide analyses of selection signatures remain scarce, constraining the identification of functional variants critical for both breeding and conservation. Furthermore, the magnitude of commercial introgression and its impact on indigenous traits remain poorly quantified. The balance between artificial selection for productivity and natural selection for environmental resilience in LC pigs also remains unresolved.

To address these knowledge gaps, we performed whole-genome resequencing of 120 LC pigs and integrated the data with genomes from 285 pigs representing 32 global populations, including wild boars, commercial breeds, and other Chinese local breeds. By applying multiple complementary methods to detect selection signals, we sought to: (i) characterize the genetic structure and phylogenetic placement of LC pigs; (ii) identify genomic regions subject to historical selection; and (iii) uncover functional candidate genes related to economically important traits such as body size and teat number. This work provides the first comprehensive genome-wide map of selection in LC pigs, offering insights into their domestication and evolutionary history while informing strategies for conservation and genomic improvement.

## MATERIALS AND METHODS

### Data collection and sequencing

Ear tissue samples were collected from 120 LC pigs at a breeding farm in Jiaozhou, Qingdao, China. Samples were preserved in 1.5 mL centrifuge tubes and stored at −80°C until DNA extraction. Phenotypic data, including body height, body length, and total teat number, were recorded for 61 individuals. Genomic DNA was extracted using the standard phenol–chloroform method, and quality was assessed by spectrophotometry (OD260/280 ratio: 1.8–2.0) and agarose gel electrophoresis (intact DNA fragments>50 kb). High-quality DNA samples (≥2 μg total DNA) were used for library preparation with the Watchmaker Enzymatic Fragmentation DNA Library Preparation Kit (Annoroad, Cat. No. 7K0019-096) and sequenced on the DNBSEQ-T7 platform.

Publicly resequencing data for 32 global pig breeds (n = 285) were obtained from NCBI ( https://www.ncbi.nlm.nih.gov/) (Supplement 1), including: Commercial breeds: Large White (LW, n = 10), Duroc (DUR, n = 10), Landrace (LR, n = 10), Iberian (IBE, n = 2), Pietrain (PIE, n = 10), Berkshire (BER, n = 4); Wild populations: European wild boar (EW, n = 15), Asian wild boar (AW, n = 17); Chinese indigenous breeds: Beijing Black (BJH, n = 10), Mashen (MAS, n = 6), Min (MIN, n = 10), Nanyang Black (NY, n = 5), Hetaodaer (HTDE, n = 10), Bama Xiang (BMX, n = 6), Diannan Small-ear (DNXE, n = 10), Baoshan (BS, n = 6), Meishan (MS, n = 23), Tibetan (TT, n = 20), Yanan (YN, n = 3), Wannan Black (WNH, n = 10), Erhualian (EHL, n = 17), Jiangquhai (JQH, n = 3), Jinhua (JH, n = 9), Daweizi (DWZ, n = 8), Neijiang (NJ, n = 9), Tongcheng (TC, n = 6), Wuzhishan (WZS, n = 5), Debao (DB, n = 7), Luchuan (LUC, n = 6), Rongchang (RC, n = 10), Tiegu (TG, n = 7), and Wujin (WJ, n = 2).

### Variant calling and quality control

Raw data were first inspected with FastQC (v0.11.9) [9] and processed with Trimmomatic (v0.38) [10] under the following criteria: removal of adapter sequences; trimming of bases with Phred scores <3 at read termini; sliding-window filtering (4 bp window, average quality threshold = 15); and discarding of reads shorter than 36 bp after trimming. Clean reads were aligned to the *Sus scrofa* reference genome (v11.1) using BWA-MEM [11].

Aligned BAM files were sorted with SAMtools (v1.6) [12]. PCR duplicates were identified and removed using GATK MarkDuplicates (v4.3.0.0) [13]. Variant calling was performed using GATK HaplotypeCaller with SelectVariants, and SNPs were filtered with the following thresholds: QD<2.0, FS>60, SOR>3.0, MQ<40, MQRankSum<−12.5, and ReadPosRankSum<−8.0. Only autosomal, biallelic SNPs with minor allele frequency (MAF) ≥5% and missing rate ≤10% were retained using VCFtools (v0.1.16) [14].

### Population structure analyses

Genetic relationship matrices (GRM) were constructed with GCTA (v1.94.0) [15] using the --make-grm option. The GRM served as the basis for principal component analysis (PCA) to assess population stratification. Population stratification was evaluated by PCA and visualized in R (v4.2.3) with ggplot2 [16]. Identity-by-state (IBS) distances were computed with PLINK, and a Neighbor-Joining (NJ) tree was generated using MEGA (v11.0) [17] and annotated in iTOL ( https://itol.embl.de/). Population admixture was estimated using ADMIXTURE (v1.3.0) [18] with the number of ancestral clusters (K) ranging from 2 to 9. The optimal K value was determined by the minimum cross-validation error. Historical gene flow was analyzed using TreeMix ( https://bitbucket.org/nygcresearch/treemix/wiki/Home) with linkage disequilibrium (LD)-pruned autosomal SNPs, testing 0–5 migration edges and using Asian wild boar as the outgroup. The analysis included LC pigs, Asian wild boar, and six commercial breeds (Large White, Duroc, Landrace, Iberian, Pietrain, and Berkshire).

### Genetic diversity analyses

Runs of homozygosity (ROH) were detected using PLINK (v1.90) [19] with the following parameters: sliding window size of 50 SNPs, minimum ROH length = 500 kb, ≥50 consecutive homozygous SNPs, and ≤5% heterozygous calls per window. LD decay was analyzed with PopLDdecay (v3.42) [20] using default settings. Pairwise LD coefficients (r^2^) were calculated across autosomal SNPs to evaluate haplotype structure and recombination patterns. Nucleotide diversity (π) was calculated using VCFtools (v0.1.16) [14]. To compare the π values of the LC against other pig breeds, Wilcoxon rank-sum tests were performed in R with statistical significance defined at p<0.05.

### Detection of selection signatures

Genomic regions under selection were identified using four complementary approaches. Firstly, population differentiation was quantified using Fixation Index (FST) in 50-kb windows (20-kb step size). Secondly, the log-transformed nucleotide diversity (θπ) ratios calculated in 50-kb windows. Thirdly, the Cross-Population Composite Likelihood Ratio (XP-CLR) were calculated in 50-kb windows. Neutrality tests were performed using Tajima’s D statistic in 50-kb windows with VCFtools (v0.1.16) [14]. Candidate regions were defined as overlapping windows within the top 5% across multiple methods.

### Functional annotation

Genome wide SNPs and candidate genomic regions (top 5% signals) were annotated using ANNOVAR (v2020-06-08) [21]. Pig quantitative trait loci (QTL) within these regions were retrieved from Pig QTLdb ( https://www.animalgenome.org/cgi-bin/QTLdb/SS/index). Gene Ontology (GO) enrichment and Kyoto Encyclopedia of Genes and Genomes (KEGG) pathway analyses were conducted with g:Profiler [22] and validated using the OmicStudio platform ( https://www.bioinformatics.com.cn). Spatiotemporal expression of candidate genes across porcine tissues was explored via the PigGTEx database ( http://piggtex.farmgtex.org/). Coding sequences (CDS) were extracted with gffread (v0.12.7) [23], protein structures were predicted with AlphaFold3 [24], and structural visualization was performed using PyMOL (v3.0.4) [25].

## RESULTS

### Genomic variation landscape of Licha black pig

**Figure 1:**
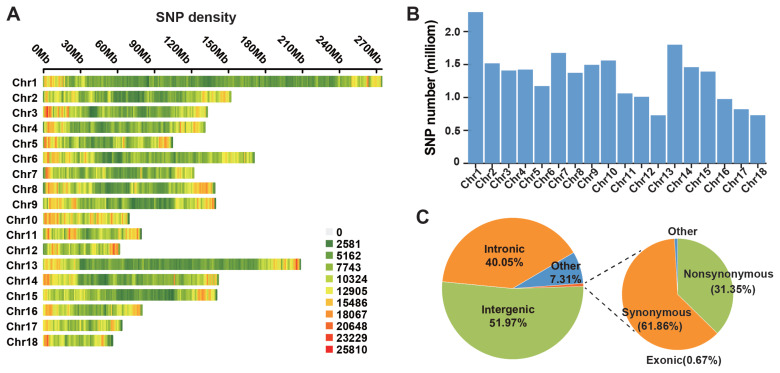
Genome-wide autosomal SNP distribution in Licha black pigs. (A) Chromosomal density of all detected SNPs. The color gradient represents the density of SNPs per megabase (SNPs/Mb), ranging from 0 (grey, low density) to 25,810 (red, high density). (B) Distribution of all detected SNP number per chromosome. (C) Functional annotation profile categorizing SNPs by genomic context and variant type.

Whole-genome resequencing of 120 LC pigs was performed at an average coverage depth of 10×. Following standard quality control, we identified a total of 19,627,493 high-quality autosomal SNPs. As shown in Figure 1A, these variants were unevenly distributed across the 18 autosomes of the *Sus scrofa* reference genome (Sscrofa11.1 assembly). Chromosome 1 exhibited the highest SNP density (1,822,690), followed by chromosomes 6 (1,495,582) and 13 (1,489,892), whereas chromosome 18 contained the fewest SNPs (576,457) (Figure 1B). Functional annotation using ANNOVAR revealed that 8,821,110 were successfully annotated. Among these, 51.97% localized to intergenic regions, 40.05% to intronic regions, and 0.67% to exonic regions. A detailed analysis of the 58,762 exonic SNPs showed that 21,948 (31.35%) were nonsynonymous, 36,349 (61.86%) were synonymous, and 465 (0.79%) were stop-gain or stop-loss mutations (Figure 1C).

### Population structure and genomic diversity of Licha black pig

**Figure 2:**
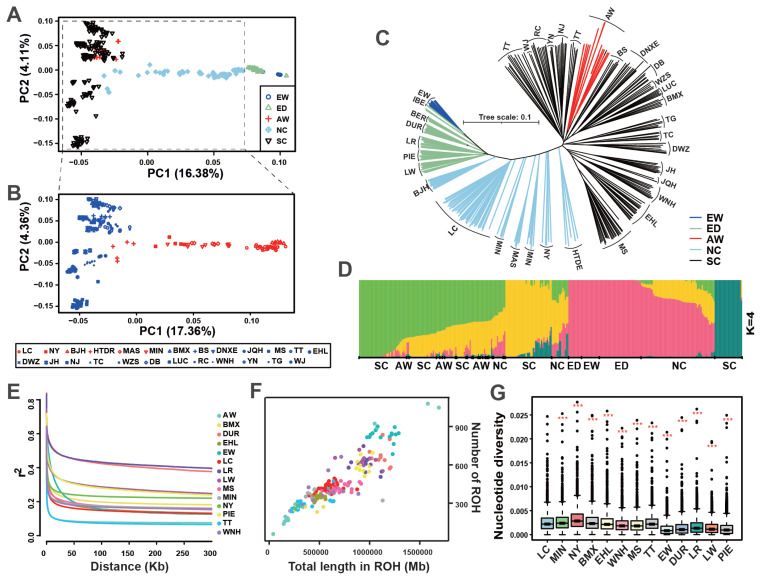
Population genetic structure and genomic diversity of Licha black pigs. (A) Principal component analysis (PCA) of global pig populations. Populations include European wild boars (EW), European-derived commercial breeds (ED, including Large White, LW; Duroc, DUR; Landrace, LR; Iberian, IBE; Pitland, PIE; Berkshire, BER), Asian wild boars (AW), Northern Chinese breeds (NC, including Beijing Black, BJH; Mashen, MAS; Min, MIN; Nanyang Black, NY; Licha Black, LC; Hetaodaer, HTDE), and Southern Chinese breeds (SC, Bama Xiang, BMX; Diannan Small-ear, DNXE; Baoshan, BS; Meishan, MS; Wuzhi Mountain, WZS; Debao, DB; Luchuan, LUC; Rongchang, RC; Tiegu, TG; Wujin, WJ). (B) PCA of indigenous Chinese pig breeds, highlighting the NC (red) and SC (blue) clusters. (C) Neighbor-joining phylogenetic tree of global pig breeds. (D) Ancestry admixture proportions of global pig breeds at K = 4. (E) Linkage disequilibrium (LD) decay profiles. (F) Runs of homozygosity (ROH) distributions. (G) Nucleotide diversity (π) across populations. Statistical significance between LC and other pig breeds was assessed by the Wilcoxon rank-sum test (*** p<0.001).

Beyond the 120 LC pigs, we analyzed 285 individuals representing 32 breeds, including commercial lines (n = 46), European wild boars (n = 15), Asian wild boars (n = 17), and 24 Chinese indigenous breeds (n = 207) (Supplement 2). PCA revealed distinct phylogeographic clustering among global populations, with a clear divergence between European and Asian lineages (Figure 2A). Within China, breeds were subdivided into northern (NC) and southern (SC) clusters along the Qinling-Huaihe boundary (Figure 2B).

Notably, although LC was geographically located north of this boundary, it exhibited a closer genetic relationship to European pig populations in both PCA and NJ tree analyses (Figures 2A–2C). This finding was further supported by admixture analysis at K = 4, which showed a population structure consistent with the PCA and phylogenetic topology (Figure 2D). TreeMix analysis further confirmed the genetic divergence between LC and commercial breeds, while also detecting a historic gene flow event from Duroc to LC (Supplement 3).

LD decay in LC between was intermediate between intensively selected European commercial breeds and minimally selected Asian wild boars. LC exhibited slower LD decay compared with some other Chinese indigenous breeds, reflecting moderate anthropogenic selection (Figure 2E). This pattern was corroborated by ROH analysis, in which LC showed an intermediate ROH level, indicating a moderate inbreeding level relative to Asian wild boars and European commercial pigs (Figure 2F). Nucleotide diversity analysis confirmed significant differences between LC and other breeds (Wilcoxon rank-sum test, all p<0.001). LC exhibited an intermediate π value (0.0025±0.0000), lower than northern Chinese breeds (e.g., NY: 0.0031±0.0000) but higher than southern Chinese (e.g., WNH: 0.0020±0.0000) and European commercial breeds (e.g., PIE: 0.0013±0.0000) (Figure 2G).

### Genome-wide scans reveal signatures of selection in Licha black pig

**Figure 3:**
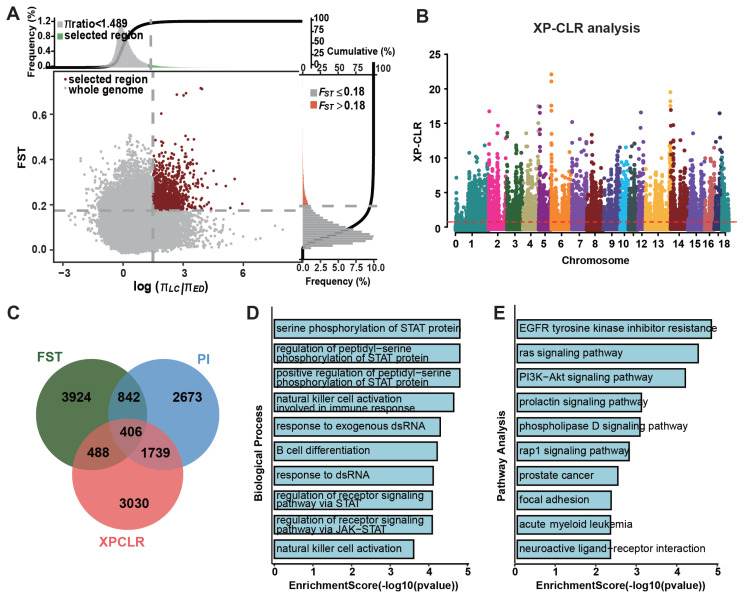
Selective sweep analysis comparing Licha black pigs with European commercial breeds. (A) Composite selection signals (FST and θπ ratio) with empirical thresholds (dashed lines: FST>0.18, θπ ratio>4.43). (B) XP-CLR scores with selection threshold (red dashed line: XP-CLR>0.75). (C) Venn diagram showing overlap of 406 genomic regions identified by all three methods. (D) Top 10 enriched Gene Ontology terms. (E) Top 10 enriched Kyoto Encyclopedia of Genes and Genomes pathways.

To uncover genomic regions underlying the adaptive and economically important traits of LC pigs, we performed genome-wide selective sweep analyses using European commercial breeds (Duroc, Landrace, and Pietrain) as the reference population. To identify genomic regions under strong selection, we integrated three approaches: FST>0.18, θπ ratio>4.43, and XP-CLR>0.75 (Figures 3A–3C). This integrative analysis yielded 406 overlapping 50-kb windows that encompass 229 candidate genes. These genes are significantly enriched in pathways related to immune adaptation, including STAT protein phosphorylation cascades (GO:0007259), natural killer cell activation (GO:0002323), and response to exogenous dsRNA (GO:0043331). Additionally, metabolic efficiency was highlighted through the enrichment of genes in the Ras/PI3K-Akt signaling pathway and prolactin-mediated lactation adaptation pathways (Figures 3D, 3E).

**Figure 4:**
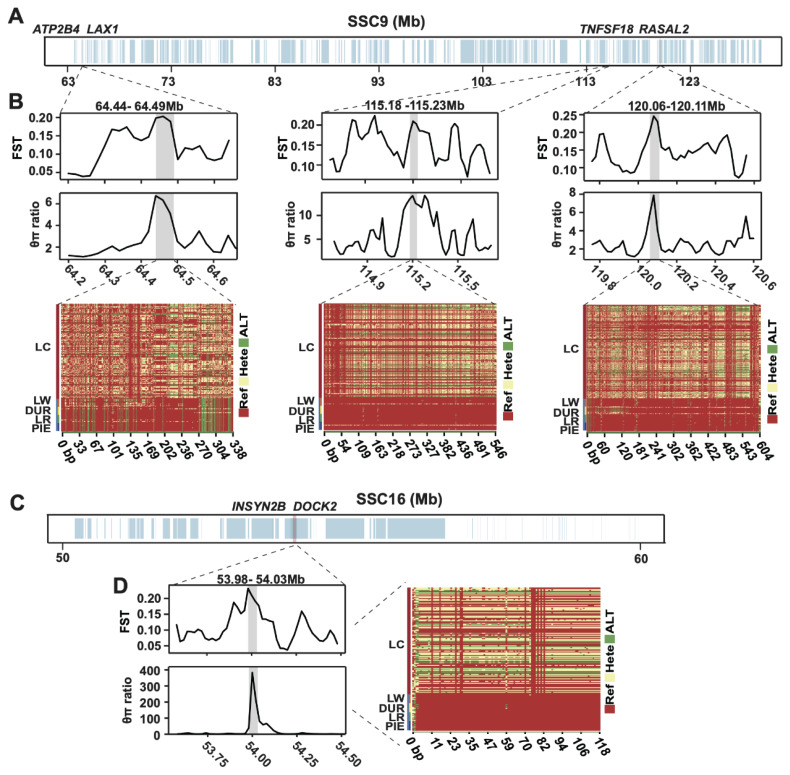
Genomic selection signatures and haplotype architecture of candidate regions. (A) *Sus scrofa* chromosome 9 (SSC9) showing gene density (blue) and candidate regions (red). (B) Corresponding selection metrics (FST top, θπ ratio middle) and haplotype heatmap (bottom). (C) *Sus scrofa* chromosome 16 (SSC16) annotation. (D) Selection metrics and haplotype patterns for SSC16.

Further refinement using Tajima’s D validation and FST/θπ ratio filtering prioritized four high-confidence loci associated with economic traits (Figures 4A–4D). On Sus scrofa chromosome 9 (SSC9), we identified: A 64.44–64.49 Mb region containing *ATP2B4* (calcium transport) and *LAX1* (immune cell migration), overlapping a QTL for average daily gain (PigQTLdb: 64,487,877–881 bp); A 115.18–115.23 Mb interval harboring *TNFSF18* (immune tolerance); A 120.06–120.11 Mb segment featuring *RASAL2* (adipogenesis/muscle development) near a multi-breed health QTL (PigQTLdb: 120,102,471–475 bp). On SSC16, a 53.98–54.03 Mb region contained INSYN2B and spermatogenesis gene *DOCK2*, coinciding with a Large White growth QTL (PigQTLdb: 53,995,793–797 bp). Haplotype fixation patterns confirmed selection pressure on these trait-associated loci. Spatiotemporal expression profiling via PigGTEx revealed biologically relevant patterns: *ATP2B4* and *RASAL2* showed high co-expression in lungs and spleen, suggesting synergistic roles in calcium signaling and energy metabolism. *TNFSF18* exhibited blastomere-specific elevation, indicating early immune programming functions. Tissue-restricted expression of *LAX1* (spleen), *INSYN2B* (hypothalamus and pituitary), and *DOCK2* (thymus) further supported roles in lymphocyte migration, neuroendocrine-immune crosstalk, and T-cell development, respectively (Supplement 4).

### Genomic loci associated with growth and reproductive traits in Licha black pig

**Figure 5:**
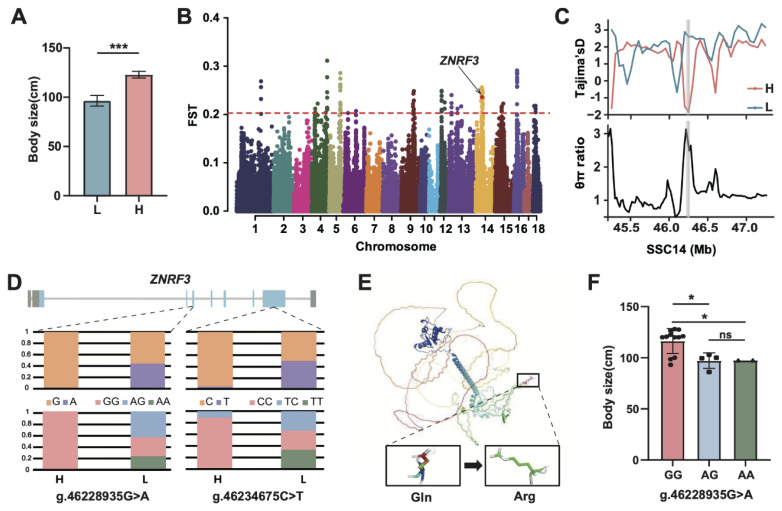
Genomic region associated with body size variation of Licha black pigs. (A) Body size index comparison between extreme phenotype groups (high: H, n = 9; low: L, n = 9). (B) Genome-wide FST distribution. (C) Tajima’s D (top) and θπ ratio (bottom) at SSC14:46.22–46.29 Mb. (D) Allele (top) and genotype (bottom) frequencies for ZNRF3 variants. (E) Predicted structural impact of g.46228935G>A (Gln**→**Arg) substitution. (F) Association between g.46228935G>A genotypes and body size index. * p<0.05, *** p<0.001.

Body height and length were measured in 61 LC. Individuals with extreme phenotypes were selected based on a composite body size index, which defined as (height+length)/2, comprising high-body size (H, n = 9) and low-body size (L, n = 9) groups (Figure 5A). Genome-wide selection scans identified 100 candidate regions under positive selection (top 0.5% FST, threshold>0.35). To refine these regions, complementary selection signatures (Tajima’s D and θπ ratio) were analyzed (Figures 5B, 5C). This confirmed a single high-confidence region on SSC14 (46.22–46.29 Mb) overlapping the *ZNRF3* locus. Within exonic sequences, two mutations were detected: g.46228935G>A and g.46234675C>T (Figure 5D). Allele frequencies at these sites showed obvious difference with body size gradients between H and L groups. Genotyping revealed dose-dependent effects: the major alleles decreased progressively across diminishing body size classes. Protein structural modelling predicted that g.46228935G>A causes a Gln→Arg substitution (Figure 5E), while g.46234675C>T is a synonymous variant. In the population, individuals homozygous for the G allele at g.46228935G>A exhibited significantly larger body size index (116.5 cm) than AG (97.1 cm) and AA (97.5 cm) genotypes (Figure 5F).

**Figure 6:**
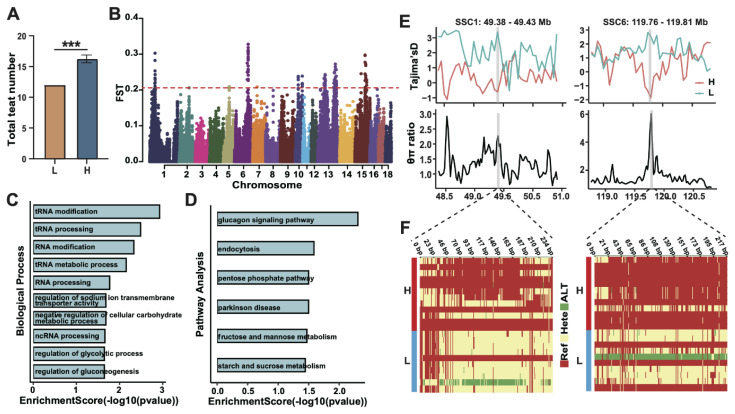
Genomic region associated with teat number variation of Licha black pigs. (A) Teat number comparison between extreme groups (high: H, n = 12; low: L, n = 10). (B) Genome-wide FST distribution. (C, D) Enriched GO terms and KEGG pathways for candidate genes. (E) Tajima’s D (top) and θπ ratio (bottom) signals across selective regions. (F) Haplotype heatmaps for candidate regions. *** p<0.001. GO, Gene Ontology; KEGG, Kyoto Encyclopedia of Genes and Genomes.

For teat number variation, phenotypic data from the same cohort were stratified into low-teat (L, n = 10) and high-teat (H, n = 12) groups (Figure 6A). FST-based selection scans (H group *vs*. L group) identified 100 top candidate regions (Figure 6B). Annotation revealed 39 candidate genes, which showed significant enrichment in biological processes including tRNA modification (GO:0006400), tRNA processing (GO:0008033), and RNA modification (GO:0009451) (Figure 6C). KEGG analysis highlighted enrichment in glucagon signalling, endocytosis, and the pentose phosphate pathway (Figure 6D). Additional validation using Tajima’s D and θπ ratio narrowed these to two high-confidence regions: SSC1:49.38–49.43 Mb (*ADGRB3*) and SSC6:119.76–119.83 Mb (*RPRD1A*) (Figure 6E). Haplotype analyses demonstrated distinct clustering between high- and low-teat groups, consistent with phenotype-associated haplotype differentiation (Figure 6F).

## DISCUSSION

Our genome-wide analysis of LC pigs provides critical insights into the genetic mechanisms underlying their distinctive traits while contextualizing their evolutionary relationship with global porcine populations. The observed genetic divergence between northern and southern Chinese breeds along the Qinling-Huaihe line, reinforcing the role of geographic barriers in shaping porcine biodiversity. Notably, LC showed moderate genetic affinity to European commercial breeds, potentially reflecting documented historical crossbreeding during breed improvement efforts. Despite this, LC retains higher genetic diversity than southern Chinese breeds and European commercial pigs, indicating resilience to genetic erosion-a crucial feature for its conservation status as a provincial-level protected genetic resource. The slow LD decay and low inbreeding coefficients further support its value as a genetic reservoir for adaptive traits, aligning with its reputation for superior environmental adaptation [26].

Selective sweep analysis against European commercial breeds identified six key genes enriched in pathways fundamental to LC’s economically valuable traits. *ATP2B4* encodes a P-type Ca^2+^-ATPase essential for cytoplasmic calcium clearance through active transmembrane extrusion. This gene’s selection correlates with LC’s muscular development [27] and may underlie growth-related QTLs. Notably, *ATP2B4* deficiency causes impaired sperm motility and male infertility in mice [28], with differential expression in bovine semen serving as a fertility biomarker [29]. *TNFSF18* belongs to the tumor necrosis factor (TNF) superfamily, which has been identified as key pathogenic cytokines mediating apoptosis in the pathogenesis of African swine fever [30]. *RASAL2* functions as a RAS-specific GTPase-activating protein that modulates adipogenesis through MAPK signalling [31]. Its selection corresponds with enhanced lipid deposition in LC [32], an adaptation to nutrient-scarce environments. This locus further associates with body size regulation in livestock [33]. *DOCK2*, a cytokine-dependent GEF, exhibits methylation patterns linked to male fertility regulation in cattle [34]. Its association with ram ejaculate concentration [35], supports its role in LC’s reproductive superiority. These loci exhibit near-fixation in LC but remain polymorphic in commercial pigs, highlighting divergent selection pressures between intensive production systems and the lower-input systems where LC evolved.

The stratification analysis based on body size traits identified *ZNRF3* as a strongly selected gene within high confidence sweep regions. This finding provides mechanistic support for LC’ distinctive skeletal development. *ZNRF3* encodes an E3 ubiquitin ligase that negatively regulates Wnt/β-catenin signaling-a pathway indispensable for osteogenesis and limb patterning [36]. Our results align with mammalian models demonstrating that *ZNRF3-RSPO2-RNF43* tripartite complexes establish morphogen gradients governing limb bud differentiation. In LC, selective pressure on *ZNRF3* likely fine-tunes skeletal proportionality, potentially explaining their characteristic stature. Notably, comparative genomic studies in European commercial breeds show divergent *ZNRF3* haplotype frequencies, suggesting independent selection trajectories for body conformation traits across genetic lineages. For teat number traits, *ADGRB3* emerged as a prime candidate gene. As an adhesion G protein-coupled receptor (GPCR), *ADGRB3* modulates cell adhesion and G-protein-coupled signaling cascades relevant to mammary morphogenesis. Our data extend prior GWAS evidence linking *ADGRB3* to mammary conformation in dairy cattle [37]. Its conserved role across species is striking. *ADGRB3* variants associate with ovarian follicle development in chickens [38], and sperm-egg fusion competence in mammals [39]. Epigenetic regulation of *ADGRB3* enhances hypoxia tolerance via angiogenesis modulation [40]. In LC, *ADGRB3* selection may coordinate teat placement patterning and mammary gland vascularization-critical for the breed’s renowned high litter size and maternal efficiency.

These results provide a roadmap for marker-assisted breeding to enhance LC’s productivity while preserving its genetic distinctiveness. The identified variants serve as candidates for introgression into commercial lines to improve resilience and reproductive efficiency. While our multi-method approach strengthened signal reliability, the moderate sample size for trait-stratified analyses warrants validation in larger cohorts. Functional validation of *ZNRF3* and *ADGRB3* variants using CRISPR models would clarify their causal roles. Additionally, epigenetic and transcriptomic profiling could reveal regulatory layers beyond sequence variation.

## CONCLUSION

This study delineates the genomic architecture of LC, elucidating the genetic foundations of their adaptive and economically valuable traits. LC exhibits northern Chinese ancestry with European introgression, retaining high diversity and adaptive potential. Compared to European commercial breeds, LC shows strong selection signatures in metabolic efficiency genes (*ATP2B4* and *RASAL2*), immune adaptation loci (*TNFSF18* and *LAX1*), reproductive fitness regulators (*DOCK2*). Body size stratification identified *ZNRF3* (SSC14:46.22–46.29 Mb) as a key determinant, where the g.46228935G>A (Gln→Arg) mutation may affect body size of LC. Teat number analysis prioritized *ADGRB3* (SSC1:49.38–49.43 Mb), with haplotypes significantly segregating between high/low-teat groups. These findings bridge functional genomics with breeding applications, offering molecular targets to enhance productivity. By integrating evolutionary history with trait-specific selection patterns, this work providing a roadmap for leveraging indigenous genetic resources in precision breeding and conservation strategies.

## Data Availability

Whole-genome sequence data generated in this study are available in the China National Centre for Bioinformation (CNCB) under accession number PRJCA043108. Public dataset accession numbers are provided in Supplement 1.

## SUPPLEMENTARY MATERIAL

**Supplement 1**. Details of publicly available resequencing data for 32 global pig breeds (n = 285).

**Supplement 2**. Geographical distribution of sampled pig populations.

**Supplement 3**. Licha Black pig population history inference using TreeMix.

**Supplement 4**. Tissue-specific expression profiles of candidate genes.
