# Supplementary material for: Genomic signatures of selection reveal genetic mechanisms underlying economic traits in Licha black pigs
Source: Anim Biosci. 2025 Dec 3;39(5):250712. doi: 10.5713/ab.250712 (PMC13175055; doi:10.5713/ab.250712)
Supplement: Supplementary file 1 [file ab-250712-Supplement-1.pdf]

Supplement 1. Details of publicly available resequencing data for 32 global pig breeds (n = 285)

Breed	Abbreviation	Sample ID	BioProject accession	Gender	Data size
Asian wild	AW	AW396	PRJEB1683	NA	9.77Gb
Asian wild	AW	AW411	PRJEB1683	NA	14.95Gb
Asian wild	AW	AW414	PRJEB1683	NA	11.99Gb
Asian wild	AW	AW417	PRJEB1683	NA	11.01Gb
Asian wild	AW	AW421	PRJEB1683	NA	10.23Gb
Asian wild	AW	AW519	PRJEB1683	NA	9.13Gb
Asian wild	AW	AW520	PRJEB1683	NA	12.85Gb
Asian wild	AW	AW521	PRJEB1683	NA	11.09Gb
Asian wild	AW	AW613	PRJNA305081	NA	13.58Gb
Asian wild	AW	AW614	PRJNA305081	NA	15.48Gb
Asian wild	AW	AW615	PRJNA305081	NA	15.99Gb
Asian wild	AW	AW815	PRJEB9922	NA	23.47Gb
Asian wild	AW	AW816	PRJEB9922	NA	35.14Gb
Asian wild	AW	AW820	PRJEB9922	NA	21.47Gb
Asian wild	AW	AW821	PRJEB9922	NA	36.24Gb
Asian wild	AW	AW822	PRJEB9922	NA	40.04Gb
Asian wild	AW	AW823	PRJEB9922	NA	39.42Gb
Bama Xiang	BMX	bamaxiang8127	PRJNA213179	NA	44.59Gb
Bama Xiang	BMX	bamaxiang8128	PRJNA213179	NA	48.12Gb
Bama Xiang	BMX	bamaxiang8129	PRJNA213179	NA	42.46Gb
Bama Xiang	BMX	bamaxiang8130	PRJNA213179	NA	46.65Gb
Bama Xiang	BMX	bamaxiang8131	PRJNA213179	NA	46.07Gb
Bama Xiang	BMX	bamaxiang8132	PRJNA213179	NA	45.81Gb
Baoshan	BS	baoshan488	PRJNA398176	Pooled	8.14Gb
Baoshan	BS	baoshan511	PRJNA398176	Pooled	9.22Gb
Baoshan	BS	baoshan513	PRJNA398176	Pooled	9.94Gb
Baoshan	BS	baoshan522	PRJNA398176	Pooled	10.63Gb
Baoshan	BS	baoshan529	PRJNA398176	Pooled	9.28Gb
Baoshan	BS	baoshan554	PRJNA398176	Pooled	10.74Gb
Beijing Black	BJH	beijinghei243	PRJNA488960	NA	21.64Gb
Beijing Black	BJH	beijinghei244	PRJNA488960	NA	19.57Gb
Beijing Black	BJH	beijinghei245	PRJNA488960	NA	19.87Gb
Beijing Black	BJH	beijinghei246	PRJNA488960	NA	17.91Gb
Beijing Black	BJH	beijinghei247	PRJNA488960	NA	23.31Gb
Beijing Black	BJH	beijinghei248	PRJNA488960	NA	21.09Gb
Beijing Black	BJH	beijinghei251	PRJNA488960	NA	22.06Gb
Beijing Black	BJH	beijinghei263	PRJNA488960	NA	21.39Gb
Beijing Black	BJH	beijinghei264	PRJNA488960	NA	20.10Gb
Beijing Black	BJH	beijinghei265	PRJNA488960	NA	21.62Gb
Berkshire	BER	Berkshire3434	PRJNA309108	NA	32.12 Gb
Berkshire	BER	Berkshire4277	PRJNA221763	NA	8.59 Gb
Berkshire	BER	Berkshire4278	PRJNA221763	NA	6.94 Gb
Berkshire	BER	Berkshire4279	PRJNA221763	NA	7.05 Gb
Daweizi	DWZ	daweizi190	PRJNA378496	Female	9.89Gb
Daweizi	DWZ	daweizi191	PRJNA378496	Female	11.64Gb
Daweizi	DWZ	daweizi960	PRJNA531381	Female	20.71Gb
Daweizi	DWZ	daweizi962	PRJNA531381	Female	24.20Gb
Daweizi	DWZ	daweizi963	PRJNA531381	Female	22.14Gb
Daweizi	DWZ	daweizi965	PRJNA531381	Female	28,33Gb
Daweizi	DWZ	daweizi966	PRJNA531381	Female	27.57Gb
Daweizi	DWZ	daweizi967	PRJNA531381	Female	25.97Gb
Debao	DB	debao943	PRJNA531381	Female	20.44Gb
Debao	DB	debao950	PRJNA531381	Female	21.04Gb

Debao	DB	debao961	PRJNA531381	Female	19.12Gb
Debao	DB	debao972	PRJNA531381	Female	22.44Gb
Debao	DB	debao983	PRJNA531381	Female	17.86Gb
Debao	DB	debao994	PRJNA531381	Female	19.28Gb
Debao	DB	debao995	PRJNA531381	Female	19.50Gb
DiannanSmall-ear	DNXE	dnxe1989	PRJNA305975	NA	9.11 Gb
DiannanSmall-ear	DNXE	dnxe1995	PRJNA305975	NA	6.79 Gb
DiannanSmall-ear	DNXE	dnxe1996	PRJNA305975	NA	8.21 Gb
DiannanSmall-ear	DNXE	dnxe1997	PRJNA305975	NA	8.37 Gb
DiannanSmall-ear	DNXE	dnxe1998	PRJNA305975	NA	7.21 Gb
DiannanSmall-ear	DNXE	dnxe1999	PRJNA305975	NA	5.72 Gb
DiannanSmall-ear	DNXE	dnxe2000	PRJNA305975	NA	8.90 Gb
DiannanSmall-ear	DNXE	dnxe2001	PRJNA305975	NA	9.02 Gb
DiannanSmall-ear	DNXE	dnxe2002	PRJNA305975	NA	5.74 Gb
DiannanSmall-ear	DNXE	dnxe2004	PRJNA305975	NA	9.31 Gb
Duroc	DUR	Dur419	PRJEB1683	NA	10.97Gb
Duroc	DUR	Dur837	PRJEB9922	NA	39.96Gb
Duroc	DUR	Dur838	PRJEB9922	NA	35.78Gb
Duroc	DUR	Dur839	PRJEB9922	NA	21.20Gb
Duroc	DUR	Dur7862	PRJNA260763	male	29.34Gb
Duroc	DUR	Dur7863	PRJNA260763	male	29.03Gb
Duroc	DUR	Dur7864	PRJNA260763	male	29.55Gb
Duroc	DUR	Dur7865	PRJNA260763	male	29.99Gb
Duroc	DUR	Dur7866	PRJNA260763	male	24.92Gb
Duroc	DUR	Dur7867	PRJNA260763	male	26.66Gb
Erhualian	EHL	erhualian141	PRJNA488327	Female	33.63Gb
Erhualian	EHL	erhualian142	PRJNA488327	Female	34.00Gb
Erhualian	EHL	erhualian144	PRJNA488327	Female	48.44Gb
Erhualian	EHL	erhualian145	PRJNA488327	Female	34.02Gb
Erhualian	EHL	erhualian146	PRJNA488327	Female	32.63Gb
Erhualian	EHL	erhualian147	PRJNA488327	Female	33.97Gb
Erhualian	EHL	erhualian148	PRJNA488327	Female	33.10Gb
Erhualian	EHL	erhualian149	PRJNA488327	Female	31.89Gb
Erhualian	EHL	erhualian157	PRJNA488327	Female	33.78Gb
Erhualian	EHL	erhualian158	PRJNA488327	Female	33.53Gb
Erhualian	EHL	erhualian2150	NA	NA	NA
Erhualian	EHL	erhualian2151	NA	NA	NA
Erhualian	EHL	erhualian2153	NA	NA	NA
Erhualian	EHL	erhualian2154	NA	NA	NA
Erhualian	EHL	erhualian2155	NA	NA	NA
Erhualian	EHL	erhualian9711	NA	NA	NA
Erhualian	EHL	erhualian9773	NA	NA	NA
European wild	EW	EW403	PRJEB1683	NA	12.60Gb
European wild	EW	EW514	PRJEB1683	NA	12.85Gb
European wild	EW	EW516	PRJEB1683	NA	9.57Gb
European wild	EW	EW518	PRJEB1683	NA	12.11Gb
European wild	EW	EW522	PRJEB1683	NA	11.52Gb
European wild	EW	EW523	PRJEB1683	NA	10.88Gb
European wild	EW	EW524	PRJEB1683	NA	12.68Gb
European wild	EW	EW525	PRJEB1683	NA	12.11Gb
European wild	EW	EW526	PRJEB1683	NA	10.08Gb
European wild	EW	EW877	PRJEB9922	NA	45.42Gb
European wild	EW	EW879	PRJEB9922	NA	34.40Gb
European wild	EW	EW884	PRJEB9922	NA	34.79Gb
European wild	EW	EW885	PRJEB9922	NA	32.06Gb

European wild	EW	EW887	PRJEB9922	NA	29.19Gb
European wild	EW	EW888	PRJEB9922	NA	26.51Gb
Tibetan	TT	GST_2327	PRJNA186497	NA	10.25 Gb
Tibetan	TT	GST_2339	PRJNA186497	NA	8.08Gb
Tibetan	TT	GST_2340	PRJNA186497	NA	8.7Gb
Tibetan	TT	GST_2341	PRJNA550237	NA	32.25 Gb
Tibetan	TT	GST_9719	PRJNA550237	NA	32.60 Gb
Tibetan	TT	GST_9720	PRJNA550237	NA	31.01 Gb
Tibetan	TT	GST_9725	PRJNA186497	NA	7.48 Gb
Tibetan	TT	GST_9726	PRJNA550237	NA	32.56 Gb
Tibetan	TT	AT_434	NA	NA	NA
Tibetan	TT	AT_435	NA	NA	NA
Tibetan	TT	AT_436	NA	NA	NA
Tibetan	TT	AT_437	NA	NA	NA
Tibetan	TT	GT_345	NA	NA	NA
Tibetan	TT	GT_349	NA	NA	NA
Tibetan	TT	GT_360	NA	NA	NA
Tibetan	TT	GT_361	NA	NA	NA
Tibetan	TT	TT_390	NA	NA	NA
Tibetan	TT	TT_408	NA	NA	NA
Tibetan	TT	TT_409	NA	NA	NA
Tibetan	TT	TT_410	NA	NA	NA
Hetaodaer	HTDE	hetaodaer715	PRJNA213179	NA	24.19Gb
Hetaodaer	HTDE	hetaodaer716	PRJNA213179	NA	21.81Gb
Hetaodaer	HTDE	hetaodaer717	PRJNA213179	NA	21.95Gb
Hetaodaer	HTDE	hetaodaer718	PRJNA213179	NA	21.36Gb
Hetaodaer	HTDE	hetaodaer723	PRJNA213179	NA	21.29Gb
Hetaodaer	HTDE	hetaodaer724	PRJNA213179	NA	20.55Gb
Hetaodaer	HTDE	hetaodaer725	PRJNA213179	NA	21.29Gb
Hetaodaer	HTDE	hetaodaer726	PRJNA213179	NA	22.20Gb
Hetaodaer	HTDE	hetaodaer727	PRJNA213179	NA	21.35Gb
Hetaodaer	HTDE	hetaodaer728	PRJNA213179	NA	21.12Gb
Iberian	IBE	Ibe012	PRJEB9922	NA	2.94Gb
Iberian	IBE	Ibe607	PRJEB9922	NA	10.45Gb
Jiangquhai	JQH	jiangquhai_795	PRJEB9922	NA	23.34Gb
Jiangquhai	JQH	jiangquhai_796	PRJEB9922	NA	15.75Gb
Jiangquhai	JQH	jiangquhai_179	PRJEB9922	NA	10.81Gb
Jinhua	JH	jinhua374	PRJNA186497	NA	11.60Gb
Jinhua	JH	jinhua375	PRJNA186497	NA	12.39Gb
Jinhua	JH	jinhua376	PRJNA186497	NA	10.60Gb
Jinhua	JH	jinhua_1	PRJNA398176	NA	41.53Gb
Jinhua	JH	jinhua_2	PRJNA398176	NA	35.60Gb
Jinhua	JH	jinhua_3	PRJNA398176	NA	30.60Gb
Jinhua	JH	jinhua_4	PRJNA398176	NA	32.18Gb
Jinhua	JH	jinhua_5	PRJNA398176	NA	29.43Gb
Jinhua	JH	jinhua_6	PRJNA398176	NA	33.98Gb
Landrace	LR	LR426	PRJEB9922	NA	13.62Gb
Landrace	LR	LR850	PRJEB9922	NA	25.95Gb
Landrace	LR	LR851	PRJEB9922	NA	12.26Gb
Landrace	LR	Landrace1	PRJNA260763	NA	22.92Gb
Landrace	LR	Landrace2	PRJNA260763	NA	23.70Gb
Landrace	LR	Landrace3	PRJNA260763	NA	22.11Gb
Landrace	LR	Landrace4	PRJNA260763	NA	21.73Gb
Landrace	LR	Landrace5	PRJNA260763	NA	21.88Gb
Landrace	LR	Landrace6	PRJNA260763	NA	21.51Gb

Landrace	LR	Landrace7	PRJNA260763	NA	22.00Gb
Large White	LW	LW402	PRJEB1683	NA	8.26Gb
Large White	LW	LW406	PRJEB1683	NA	8.96Gb
Large White	LW	LW413	PRJEB1683	NA	9.17Gb
Large White	LW	LW415	PRJEB1683	NA	9.55Gb
Large White	LW	LW422	PRJEB1683	NA	10.16Gb
Large White	LW	LW431	PRJEB1683	NA	9.30Gb
Large White	LW	LW389	PRJEB1684	NA	23.99Gb
Large White	LW	LW1135	PRJNA260763	NA	28.68Gb
Large White	LW	LW1136	PRJNA260763	NA	20.27Gb
Large White	LW	LW1137	PRJNA260763	NA	22.48Gb
Luchuan	LUC	luchuan8087	PRJNA213179	NA	41.32Gb
Luchuan	LUC	luchuan8088	PRJNA213179	NA	46.56Gb
Luchuan	LUC	luchuan8089	PRJNA213179	NA	47.12Gb
Luchuan	LUC	luchuan8090	PRJNA213179	NA	45.05Gb
Luchuan	LUC	luchuan8091	PRJNA213179	NA	49.33Gb
Luchuan	LUC	luchuan8092	PRJNA213179	NA	42.97Gb
Mashen	MAS	mashen757	PRJNA691462	NA	34.95Gb
Mashen	MAS	mashen758	PRJNA691462	NA	20.85Gb
Mashen	MAS	mashen759	PRJNA691462	NA	22.25Gb
Mashen	MAS	mashen760	PRJNA691462	NA	22.34Gb
Mashen	MAS	mashen761	PRJNA691462	NA	35.49Gb
Mashen	MAS	mashen762	PRJNA691462	NA	28.90Gb
Meishan	MS	meishan188	PRJNA378496	Female	8.62Gb
Meishan	MS	meishan624	PRJNA378496	Female	9.49Gb
Meishan	MS	meishan628	PRJNA378496	Female	9.14Gb
Meishan	MS	meishan726	PRJNA691462	NA	26.35Gb
Meishan	MS	meishan727	PRJNA691462	NA	27.19Gb
Meishan	MS	meishan728	PRJNA691462	NA	27.37Gb
Meishan	MS	meishan730	PRJNA691462	NA	29.90Gb
Meishan	MS	meishan731	PRJNA691462	NA	26.35Gb
Meishan	MS	meishan865	PRJNA378496	Female	10.70Gb
Meishan	MS	meishan874	PRJNA378496	Female	11.45Gb
Meishan	MS	meishan0205	NA	NA	NA
Meishan	MS	meishan0393	NA	NA	NA
Meishan	MS	meishan0428	NA	NA	NA
Meishan	MS	meishan8328	NA	NA	NA
Meishan	MS	meishan2589	NA	NA	NA
Meishan	MS	meishan2956	NA	NA	NA
Meishan	MS	meishan2978	NA	NA	NA
Meishan	MS	meishan3369	NA	NA	NA
Meishan	MS	meishan3583	NA	NA	NA
Meishan	MS	meishan3661	NA	NA	NA
Meishan	MS	meishan4163	NA	NA	NA
Meishan	MS	meishan6517	NA	NA	NA
Meishan	MS	meishan6917	NA	NA	NA
Min	Min	min074	PRJNA305081	NA	11.00Gb
Min	Min	min077	PRJNA305081	NA	13.54Gb
Min	Min	min078	PRJNA305081	NA	13.42Gb
Min	Min	min082	PRJNA305081	NA	12.30Gb
Min	Min	min083	PRJNA305081	NA	11.81Gb
Min	Min	min625	PRJNA671763	Male	18.46Gb
Min	Min	min629	PRJNA671763	Female	19.54Gb
Min	Min	min758	PRJNA305081	NA	13.78Gb
Min	Min	min876	PRJNA305081	NA	12.09Gb

Nanyang Black	NY	ny751	PRJNA691462	NA	42.59Gb
Nanyang Black	NY	ny752	PRJNA691462	NA	24.65Gb
Nanyang Black	NY	ny753	PRJNA691462	NA	26.23Gb
Nanyang Black	NY	ny754	PRJNA691462	NA	37.90Gb
Nanyang Black	NY	ny756	PRJNA691462	NA	26.06Gb
Neijiang	NJ	neijiang357	PRJNA186497	NA	10.23Gb
Neijiang	NJ	neijiang362	PRJNA186497	NA	11.23Gb
Neijiang	NJ	neijiang483	PRJNA398176	NA	8.79Gb
Neijiang	NJ	neijiang495	PRJNA398176	NA	9.67Gb
Neijiang	NJ	neijiang497	PRJNA398176	NA	9.03Gb
Neijiang	NJ	neijiang732	PRJNA691462	NA	10.27Gb
Neijiang	NJ	neijiang734	PRJNA691462	NA	3.74Gb
Neijiang	NJ	neijiang735	PRJNA691462	NA	4.17Gb
Neijiang	NJ	neijiang736	PRJNA691462	NA	3.42Gb
Piétrain	PIE	Pie397	PRJEB1683	NA	10.19Gb
Piétrain	PIE	Pie860	PRJEB1683	NA	23.60Gb
Piétrain	PIE	Pietrain1	PRJNA553106	male	42.09 Gb
Piétrain	PIE	Pietrain2	PRJNA553106	female	34.42 Gb
Piétrain	PIE	Pietrain3	PRJNA553106	female	35.13 Gb
Piétrain	PIE	Pietrain4	PRJNA553106	female	42.26 Gb
Piétrain	PIE	Pietrain5	PRJNA553106	male	40.28 Gb
Piétrain	PIE	Pietrain6	PRJNA553106	male	38.90 Gb
Piétrain	PIE	Pietrain7	PRJNA553106	male	40.07 Gb
Piétrain	PIE	Pietrain8	PRJNA553106	male	40.53 Gb
Rongchang	RC	rongchang1	PRJNA231897	female	13.42 Gb
Rongchang	RC	rongchang2	PRJNA231897	female	16.11 Gb
Rongchang	RC	rongchang3	PRJNA231897	NA	17.34 Gb
Rongchang	RC	rongchang4	PRJNA231897	NA	18.44 Gb
Rongchang	RC	rongchang5	PRJNA231897	NA	17.54 Gb
Rongchang	RC	rongchang6	PRJNA231897	female	14.68 Gb
Rongchang	RC	rongchang7	PRJNA231897	female	52.07 Gb
Rongchang	RC	rongchang8	PRJNA231897	female	23.29 Gb
Rongchang	RC	rongchang9	PRJNA231897	male	20.02 Gb
Rongchang	RC	rongchang10	PRJNA231897	female	24.09 Gb
Tiegu	TG	tiegu968	PRJNA531381	Female	23.99Gb
Tiegu	TG	tiegu969	PRJNA531381	Female	26.10Gb
Tiegu	TG	tiegu970	PRJNA531381	Female	22.68Gb
Tiegu	TG	tiegu971	PRJNA531381	Female	19.89Gb
Tiegu	TG	tiegu973	PRJNA531381	Female	19.52Gb
Tiegu	TG	tiegu974	PRJNA531381	Female	20.21Gb
Tiegu	TG	tiegu975	PRJNA531381	Female	21.96Gb
Tongcheng	TC	tongcheng553	PRJNA488960	NA	13.21Gb
Tongcheng	TC	tongcheng554	PRJNA488960	NA	12.82Gb
Tongcheng	TC	tongcheng557	PRJNA488960	NA	11.67Gb
Tongcheng	TC	tongcheng558	PRJNA488960	NA	12.14Gb
Tongcheng	TC	tongcheng563	PRJNA488960	NA	12.47Gb
Tongcheng	TC	tongcheng564	PRJNA488960	NA	10.82Gb
Wannan black	WNH	wannanhei961	PRJNA524263	Female	8.89Gb
Wannan black	WNH	wannanhei964	PRJNA524263	Female	8.49Gb
Wannan black	WNH	wannanhei965	PRJNA524263	Female	8.79Gb
Wannan black	WNH	wannanhei966	PRJNA524263	Female	7.00Gb
Wannan black	WNH	wannanhei968	PRJNA524263	Female	9.24Gb
Wannan black	WNH	wannanhei972	PRJNA524263	Female	9.96Gb
Wannan black	WNH	wannanhei973	PRJNA524263	Female	7.07Gb
Wannan black	WNH	wannanhei975	PRJNA524263	Female	9.59Gb

Wannan black	WNH	wannanhei976	PRJNA524263	Female	6.62Gb
Wannan black	WNH	wannanhei980	PRJNA524263	Female	7.90Gb
Wujin	WJ	wujin351	PRJNA186497	NA	11.00Gb
Wujin	WJ	wujin352	PRJNA186497	NA	9.78Gb
Wuzhishan	WZS	wuzhishan_073	PRJNA213179	NA	43.95Gb
Wuzhishan	WZS	wuzhishan_074	PRJNA213179	NA	44.91Gb
Wuzhishan	WZS	wuzhishan_076	PRJNA213179	NA	42.75Gb
Wuzhishan	WZS	wuzhishan_077	PRJNA213179	NA	44.17Gb
Wuzhishan	WZS	wuzhishan_078	PRJNA213179	NA	46.84Gb
Yanan	YN	yanan354	PRJNA186497	NA	8.09Gb
Yanan	YN	yanan355	PRJNA186497	NA	6.52Gb
Yanan	YN	yanan356	PRJNA186497	NA	7.86Gb
