# Supplementary figures and images for: Genomic signatures of selection reveal genetic mechanisms underlying economic traits in Licha black pigs
Source: Anim Biosci. 2025 Dec 3;39(5):250712. doi: 10.5713/ab.250712 (PMC13175055; doi:10.5713/ab.250712)

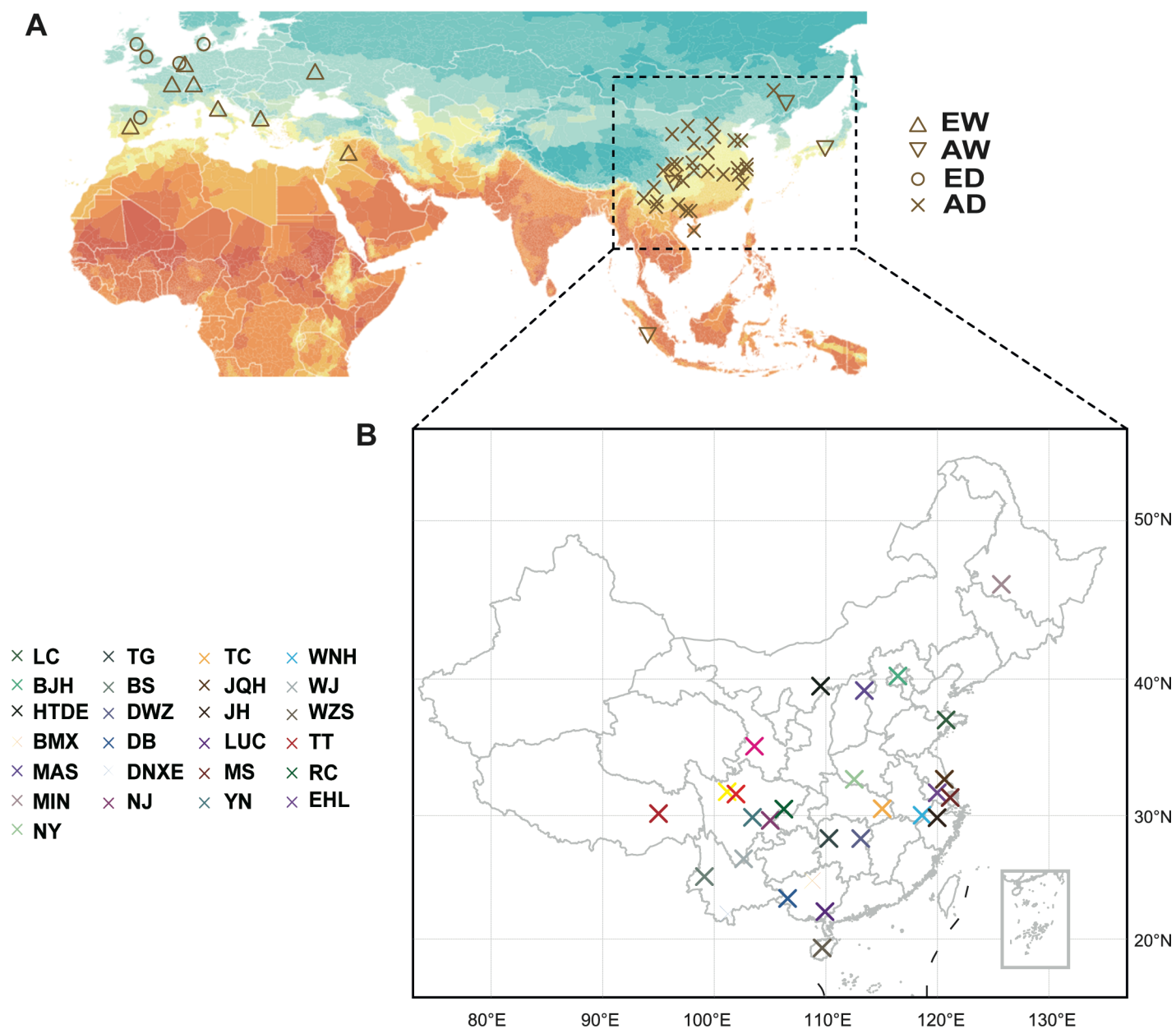

Supplement 2. Geographical distribution of sampled pig populations.

Supplement: Supplementary file 2 [file ab-250712-Supplement-2.pdf]

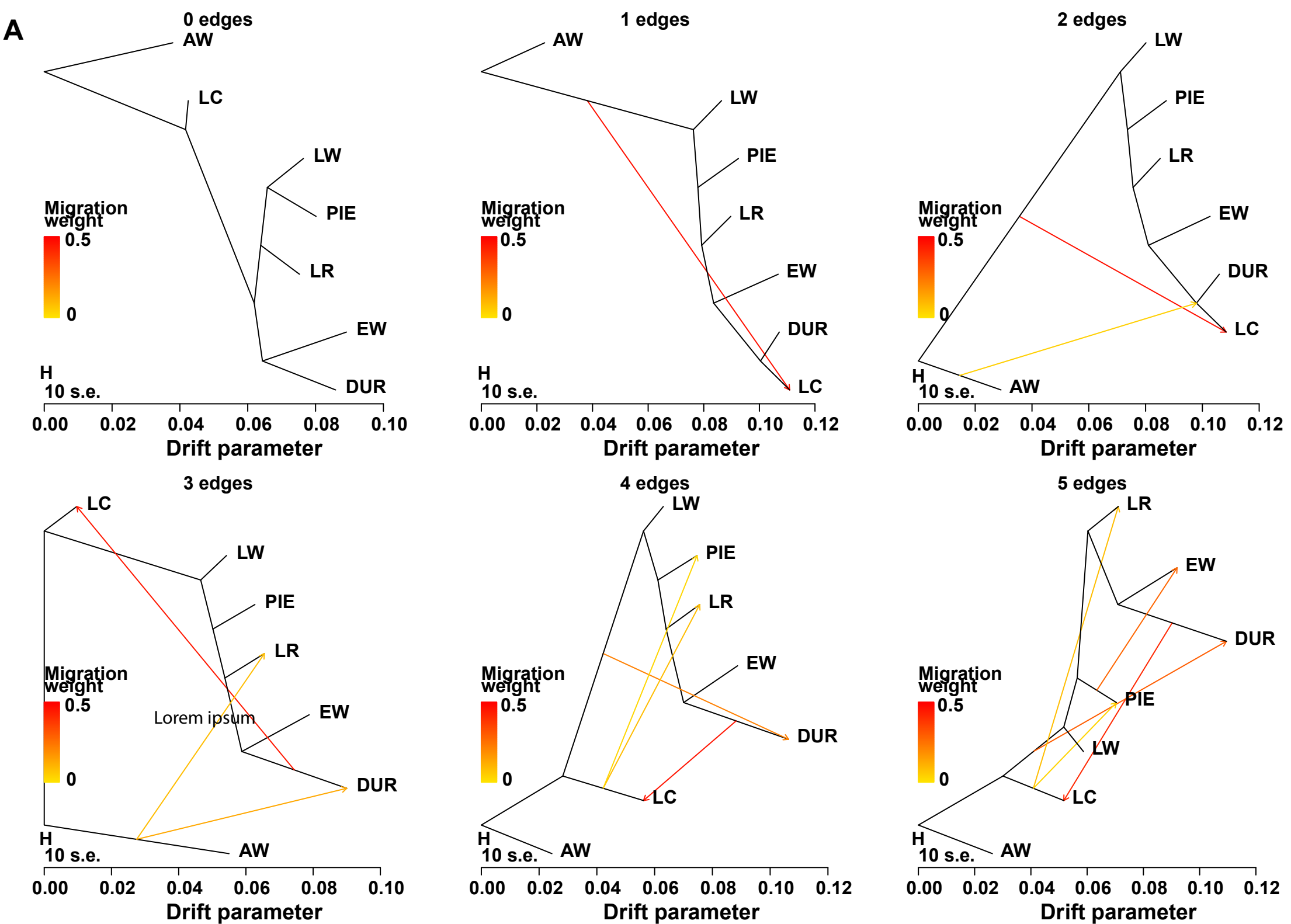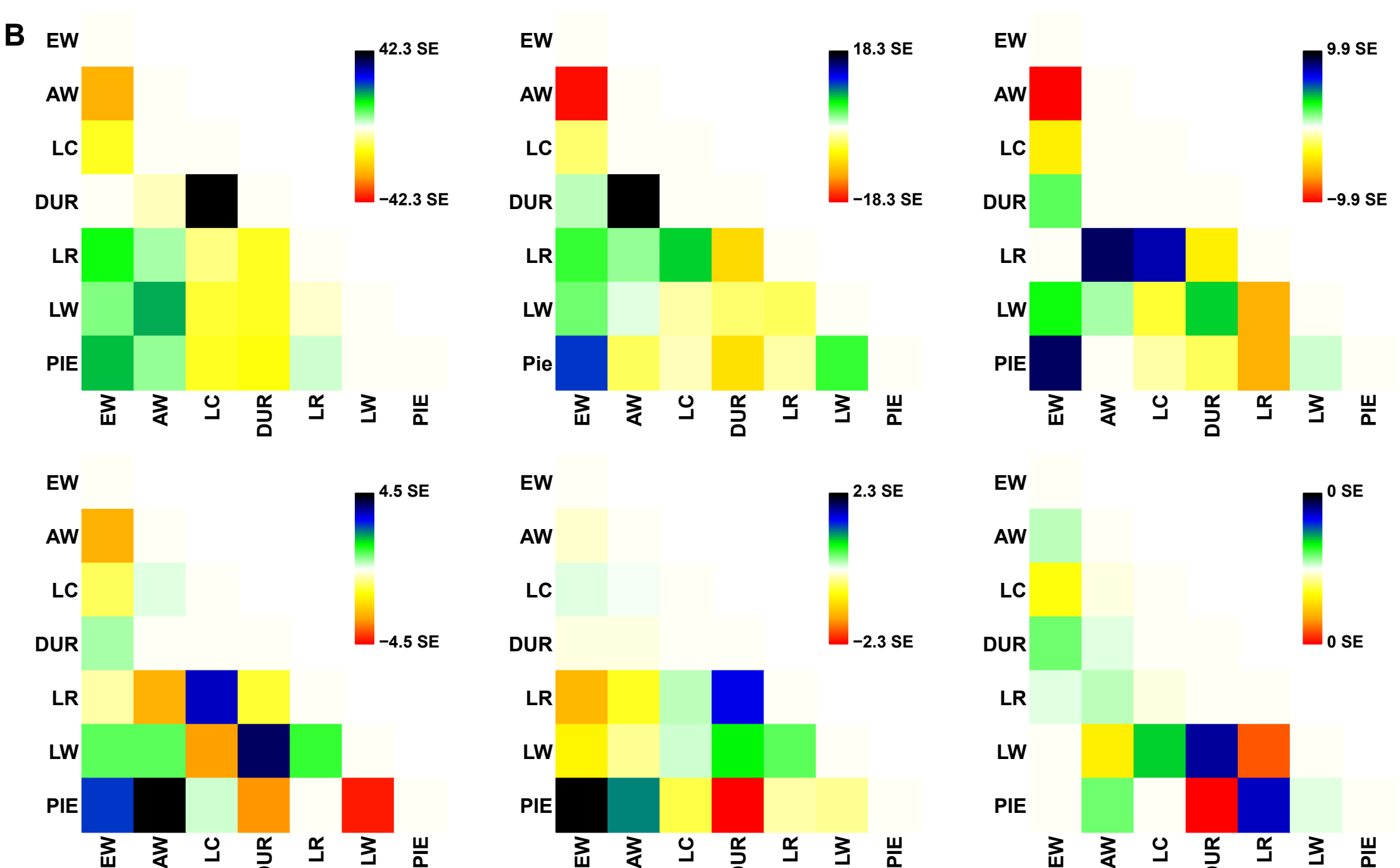

Supplement: Supplementary file 3 [file ab-250712-Supplement-3.pdf]

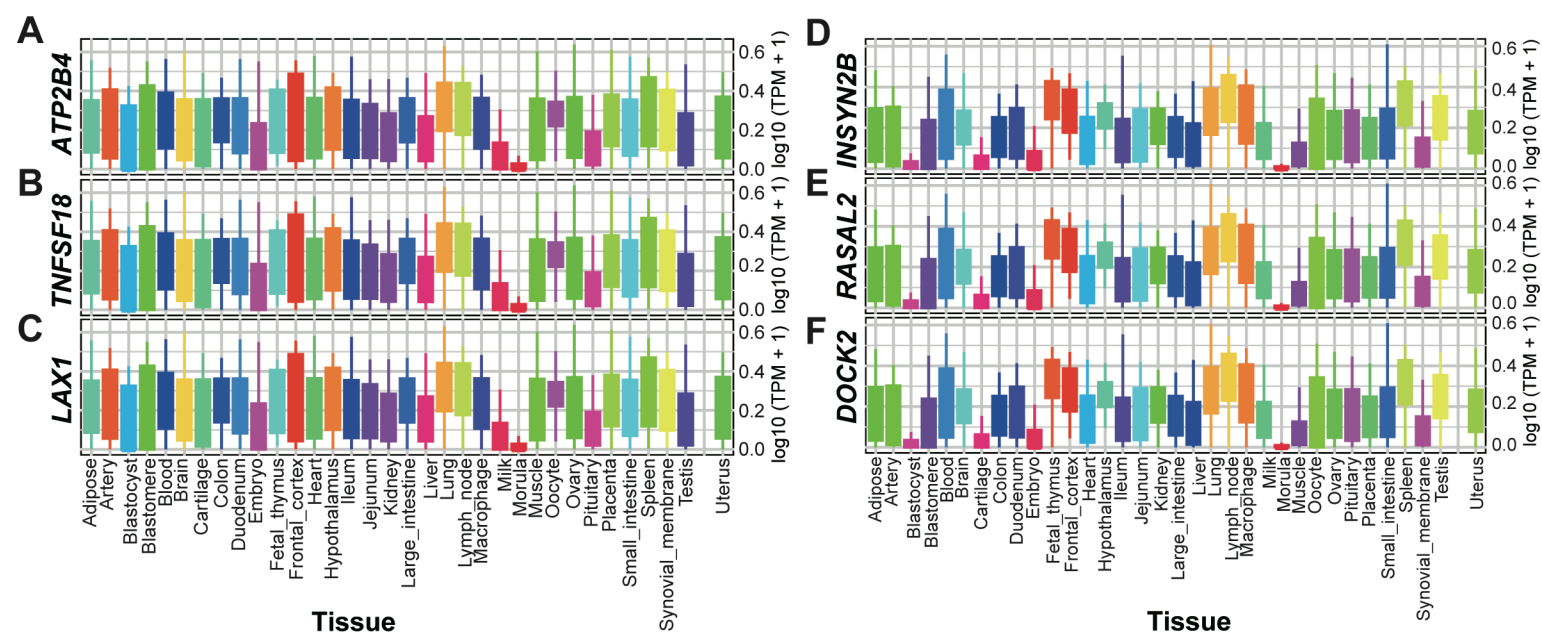

Supplement 4. Tissue-specific expression profiles of candidate genes.

Supplement: Supplementary file 4 [file ab-250712-Supplement-4.pdf]
